# Exploring and validating physicochemical properties of mangiferin through GastroPlus^®^ software

**DOI:** 10.4155/fsoa-2016-0055

**Published:** 2017-01-16

**Authors:** Rajneet Kaur Khurana, Ranjot Kaur, Manninder Kaur, Rajpreet Kaur, Jasleen Kaur, Harpreet Kaur, Bhupinder Singh

**Affiliations:** 1 University Institute of Pharmaceutical Sciences, UGC Centre of Advanced Studies, Panjab University, Chandigarh 160014, India; 2 Centre with Potential for Excellence in Biomedical Sciences, Panjab University, Chandigarh 160014, India; 3 D.A.V. College, Sector 10 D, Chandigarh 160011, India; 4 Department of Botany, G.H.G. Khalsa College, Gurusar Sadhar, Ludhiana 141104, India; 5 UGC-Centre of Excellence in Applications of Nanomaterials, Nanoparticles & Nanocomposites (Biomedical Sciences), Panjab University, Chandigarh 160014, India

**Keywords:** anticancer, antioxidants, *Mangifera indica*, P-gp substrate, simulations

## Abstract

**Aim:**

Mangiferin (Mgf), a promising therapeutic polyphenol, exhibits poor oral bioavailability. Hence, apt delivery systems are required to facilitate its gastrointestinal absorption. The requisite details on its physicochemical properties have not yet been well documented in literature. Accordingly, in order to have explicit insight into its physicochemical characteristics, the present work was undertaken using GastroPlus™ software.

**Results:**

Aqueous solubility (0.38 mg/ml), log P (-0.65), P_eff_ (0.16 × 10^-4^ cm/s) and ability to act as P-gp substrate were defined. Potency to act as a P-gp substrate was verified through Caco-2 cells, while P_eff_ was estimated through single pass intestinal perfusion studies. Characterization of Mgf through transmission electron microscopy, differential scanning calorimetry, infrared spectroscopy and powder x-ray diffraction has also been reported.

**Conclusion:**

The values of physicochemical properties for Mgf reported in the current manuscript would certainly enable the researchers to develop newer delivery systems for Mgf.

## Lay abstract

Mangiferin (Mgf), being a potent bioactive antioxidant, holds tremendous promise in the treatment of cancer, inflammation and diabetes. However, when administered orally, Mgf demonstrates poor and variable bioavailability. Accordingly, it calls for development of new drug delivery systems to improve its bioavailability. Knowledge of physicochemical attributes for a bioactive is an essential prerequisite for successful development of systems for its delivery. The current research was undertaken to explore the physicochemical attributes of Mgf and compare the same with some of those reported previously. The current research article intends to enable scientists to meet the physicochemical challenges of this bioactive and subsequently formulate it for effective drug delivery.

## Graphical abstract

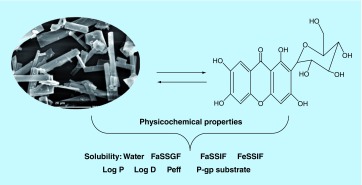

**Figure 1.**
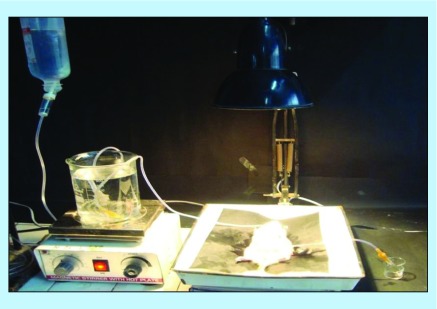
**Pictographical description of in-house assembly for performing *in situ* single pass intestinal perfusion studies.**

**Figure 2.**
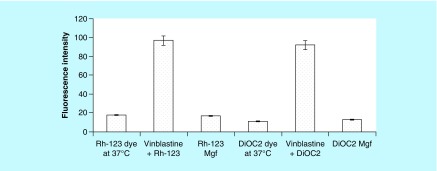
**Inhibition of mangiferin due to MDRI transporters for Rhodamine-123 loaded dye and BCRP transporters for diethyloxacarbocyanine loaded dye through low fluorescence intensity.** DiOC_2_: Diethyloxacarbocyanine; Mgf: Mangiferin; Rh-123: Rhodamine-123.

**Figure 3.**
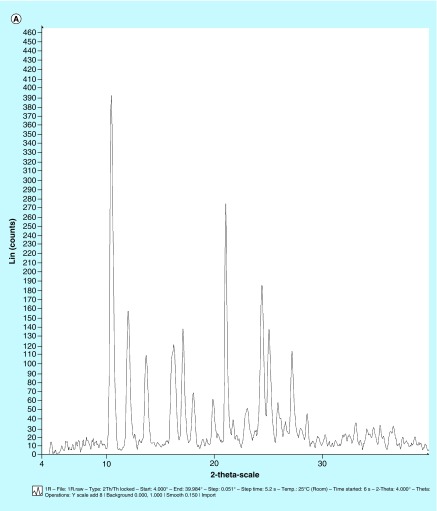

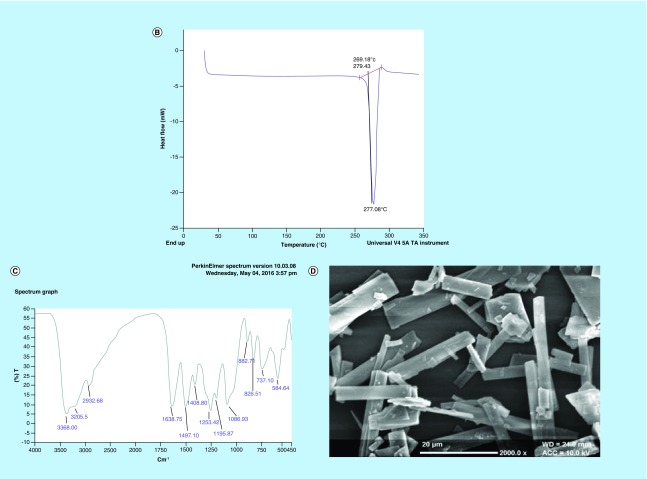
**Characterization of any bioactive helps in its proper standardization.** Hence, mangiferin was characterized for **(A)** x-ray diffraction pattern; **(B)** differential scanning calorimetry thermograms; **(C)** Fourier transform IR spectra; and **(D)** scanning electron microscopy picture (×2000).

Antioxidants are promising bioactives that play key roles in the prevention and treatment of a large number of disease states [1]. These act by scavenging free radicals, the devils underlying the cause of most deadly diseases. In this context, one of the naturally occurring polyphenolic glycosides that have lately caught the keen interest of the researchers is mangiferin (Mgf) [2]. Mgf is primarily obtained from the leaves, stem barks and fruits of *Mangifera indica*, belonging to the family Anacardiaceae. It possesses numerous activities such as being anti-inflammatory [3], antidiabetic [4], antiviral [5], analgesic [3], immunomodulatory [6] and anticancer [7]. Being a polyphenolic compound, it acts against the damage caused by oxidative stress, a leading cause of lipid peroxidation and DNA damage, and hence could combat cancer [8]. It could also be a useful therapeutic compound in alleviating neurodegenerative diseases, where oxidative stress plays a crucial role [9]. In Cuba, decoctions of Mgf are sold as nutrient supplement and phytomedicine, under the name of Vimang^®^ [10]. Owing to its reportedly remarkable therapeutic activity with hardly any reported side effects [7], Mgf has been drawing increasing attention as a potential anticancer candidate.

Mgf, however, encounters numerous challenges when administered orally, principally due to its poor solubility [11], low bioavailability [12], high hepatic first-pass metabolism [13] and high P-gp efflux [14]. Looking into these challenges, the need of the hour is to incorporate Mgf into a delivery system that has distinct potential to improve its bioavailability and, eventually, increase drug levels in systemic circulation.

Since there are a good number of studies reporting poor oral absorption of Mgf [11,15–17], there is ample scope to improve upon the formulation aspects of Mgf, provided its physicochemical attributes are known. Liu *et al*. [16] have reported that the oral absorption of Mgf from rats is quite low with bioavailability of only 1.2%, and the maximum plasma levels being quite low and inconsistent, in other words, 715.04 ± 600.14 ng/ml obtained after 0.72 h of its oral intake. Increased exposure of Mgf was found in the plasma of streptozotocin-induced diabetic rats with C_max_ and AUC_0–t_ values rising to 2.79-fold and 2.35-fold, respectively, when compared with those in the normal rats, majorly attributable to the improvement in the intestinal flora of the former. Such reports indicate the need to develop apt drug delivery formulations of Mgf with its improved biopharmaceutical potential.

Several formulation approaches have been employed for solubility enhancement of Mgf like cyclodextrin inclusion complexes [18], phospholipid complexes [19], liposomes [20], microencapsulated systems [21] and nanocapsules [22], among others. None of these techniques, however, have been found to be highly effective in enhancing the solubility and subsequent oral bioavailability. Furthermore, commercially, Mgf has so far been administered as conventional tablets, along with other antioxidants. Such combinations have proven to be of low potential to surmount the oral bioavailability problems of Mgf [23]. Primarily, low data availability on account of its inadequate fundamental physiochemical characteristic aspects has not aided researchers to formulate apt delivery systems. There is, therefore, a dire need to explore and document the physiochemical properties of such a promising antioxidant, thus facilitating the research industry to fight against deadly diseases in a more effective and safer manner.

The present research work endeavors to investigate various aspects of Mgf such as its equilibrium solubility in water, simulated gastric fluid and simulated intestinal fluid, melting point, lipophilicity as logarithm of octanol–water partition coefficient and tendency to act as a P-gp substrate, estimated through the ‘*in silico*’ approach employing GastroPlus^®^ software. This short research communication, thereby, reports the important physiochemical properties of Mgf predicted through GastroPlus^®^ *vis-à-vis* the experimentally obtained values reported in literature. Furthermore, Mgf has also been characterized through transmission electron microscopy (TEM), differential scanning calorimetry (DSC), fourier transform infrared (FTIR) and powder x-ray diffraction (P-XRD) studies. Based on the provided *in silico* and observed data, a better preview of the pharmacokinetic parameters of Mgf can be explored in clinical trials.

## Materials & methods

### Standards & reagents

Mgf was purchased from M/s Sigma-Aldrich Co. (Mumbai, India). Analytical grade ethyl acetate, acetic acid, formic acid and methanol were purchased from M/s Merck Ltd. (Mumbai, India). All other solvents and chemicals used in the studies were of analytical grade and were used as received without any further purification.

### Simulations

The *in silico* simulations on Mgf were performed using ADMET Predictor (Version 7.1.0013; Simulations Plus, Inc., CA, USA) and GastroPlus^®^ Simulation software (Version 8.6; Simulations Plus, Inc.). The simulations on Mgf were performed at Dr Reddy's laboratory, Hyderabad, India, and the results obtained have been compiled in Table 1.

### Preparation of standard solutions & TLC analysis

The stock solution was prepared by accurately weighing an amount of 10 mg of Mgf followed by dilution with methanol in a 10-ml volumetric flask to obtain the drug solution with a concentration of 1000 μg/ml. The previously validated method reported by our group for analyzing Mgf in various samples was employed using a Camag high performance thin layer chromatography (HPTLC) system equipped with a Linomat-V semi-automatic sample applicator [24]. A suitable and optimized mobile phase composition was employed, which consists of a mixture of organic and aqueous solvents *viz.* ethyl acetate, acetic acid, formic acid and water in 7:1:1:1 (v/v/v/v) ratio. Densitometric scanning of Mgf was performed at 262 nm (using a deuterium lamp) with a Camag TLC Scanner III in remission/absorption mode operated using a user-friendly WinCATS software (version 1.4.2, Muttenz, Switzerland) [10].

### Solubility studies

Equilibrium solubility is the most vital physicochemical property that reflects the bioavailability of a compound. In the current research article, solubility of the bioactive was evaluated typically in water, fasted-state simulated gastric fluid, fasted-state simulated intestinal fluid and fed-state simulated intestinal fluid by adding an excess amount of Mgf in the media, and shaken at 37 ± 1°C in a water bath shaker for 72 h. The equilibrated samples were centrifuged at 5000 r.p.m. (1398 × *g*) for 10 min to remove any undissolved drug and the supernatant was analyzed using HPTLC at a λ_max_ of 262 nm. All the media were prepared according to the composition reported by Dressman and co-workers [25].

### Melting point

A small quantity of free Mgf was placed in a thin-walled capillary tube, 10–15 cm long, closed at one end. The capillary tube containing the sample and a thermometer fixed within the melting point apparatus (Acumen Labware, Haryana, India) were heated slowly and simultaneously. The temperature range over which the melting of sample was observed was taken as its melting point [26].

### Determination of partition coefficient (log P)

Lipophilicity is a molecular property of any compound that is vital to discern the extent of distribution in the body [27]. The logarithm of octanol/water partition coefficient, in other words, log P, is the most extensively used parameter to quantify lipophilicity. The value of log P is a valuable predictor to determine the passive transport of drugs through the lipoid membranes of the human body. The value of log P of Mgf was measured using the traditional shake-flask technique at 25.0 ± 0.1°C. For this, first presaturation of the solvents (octanol and water) was conducted for 24 h [28]. Then, Mgf in a concentration of 5 mg/100 ml was added to the equilibrated mixture. After separation of the equilibrated phases by centrifugation at 10,000 r.p.m. for 5 min, the concentration of the solute was determined in the aqueous phase using HPTLC at a λ_max_ 262 nm for each sample:Equation 1
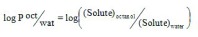



### Determination of log D

Log D, the logarithm of distribution constant or apparent partition, therefore, is a better descriptor of the lipophilicity of an ionizable compound that is likely to be charged at the physiological pH [26]. This can be determined in a similar manner to log P, but the aqueous phase, instead of being pure water, is adjusted to pH 7.4 using a physiological buffer:Equation 2
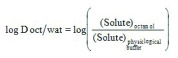



### *In situ* single pass intestinal perfusion studies

The *in situ* single pass intestinal perfusion studies were performed in a manner reported previously by our group in the past [29]. Three unisex Wistar rats (250–280 g) were fasted for at least 24 h prior to the study, while water was provided *ad libitum.* The animals were procured from University Centre Animal House, after obtaining the requisite approval from Animals Institutional Committee, Chandigarh (PU/IAEC/S/14/104). Anesthesia was induced by an intraperitoneal injection of thiopental sodium in the dose of 50 mg/kg of the body weight of the rats [29]. After making an incision in the abdomen, provisions for inlet and outlet were made at proximal part of the jejunum, 2–4 cm below the ligament of Trietz and about 10 cm distal to the first incision. Both the incised points were cannulated with polyethylene tubing and perfused with Kreb's ringer buffer (KRB) using an in-built constant infusion pump (Figure 1). The perfusate was maintained at 37 ± 1°C on a water bath. During the experiment, the rats were kept under a heating lamp and the exposed abdomen was covered with a saline-wetted cotton pad. The intestine was subsequently perfused with various formulations maintained at 37 ± 1°C at a perfusion rate of 0.2 ml/min. After attaining the steady state at 30 min, an aliquot (1 ml each) was periodically withdrawn at regular intervals of 15 min each. Diethyl ether (4 ml) was added to each perfusate sample (1 ml), and the mixture was centrifuged at 5000 r.p.m. (2795 × *g*) for 20 min. Following centrifugation, the supernatant etheral fraction (3 ml) was collected and evaporated. Finally, 1.5 ml of perfusate was added to the ethereal extract and the concentration of Mgf in the mixture was determined using HPTLC technique [24] at 254 nm by applying the principles of Analytical Quality by Design [24,30]. The value of effective permeability (P_eff_) was subsequently calculated using Equation 3 [31].Equation 3
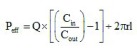



where Q is the flow rate, C_in_ and C_out_ are the respective inlet and outlet concentrations, r is the radius of the intestine and l is the length of the intestine measured after completion of perfusion [32].

### P-gp efflux assay

Furthermore, the multidrug resistance directs dye efflux assay kit (Chemicon^®^ International, CA, USA and Serologicals^®^ Corporations, GA, USA) was employed, in which the Caco-2 cells were loaded with rhodamine 123 (Rh-123) and diethyloxacarbocyanine (DiOC_2_) dyes in the presence or absence of vinblastine [33]. In order to access the functional activity of the inhibition of overexpressed MDR1, MRP1 and BCRP membrane pumps on Caco-2 cells in the presence of Mgf, the fluorescence intensity was measured in a Tecan fluorescence microplate reader (Tecan Ltd, Männedorf, Switzerland) at an excitation wavelength of 485 nm and an emission wavelength of 530 nm.

For the purpose, Caco-2 cells in the concentration of 2.5 × 10^5^ were incubated with Rh-123 alone and with vinblastine, and Rh-123-loaded Mgf at 37°C to access the inhibitory activity of Mgf on MDR1 transporters. Likewise, to estimate the ability of Mgf to act as a substrate for BCRP transportors, DiOC_2_ dye was employed. On the similar heels, Caco-2 cells (2.5 × 10^5^) were incubated with DiOC_2_ alone and with vinblastine, and DiOC_2_-loaded Mgf at 37°C.

### Statistical analysis

In order to investigate the statistical significance difference among various parameters, Student's unpaired *t*-test was applied using GraphPad Prism software ver 5.0 (M/s GraphPad Software Inc., CA, USA) [34].

### Spectroscopic, thermoanalytical & microscopic characterization

#### x-ray powder diffraction studies

The x-ray powder diffraction studies were carried out on Mgf for identifying its physical state, and the diffraction patterns of Mgf were recorded under ambient conditions on x-ray powder diffractometer (D8 Advance, Bruker, Karlsruhe, Germany) using Cu Kα radiation (= 1.54 Å) at 40 kV, 40 mA passed through a nickel filter [35].

### DSC analysis

The physical state of Mgf was also characterized using DSC Q20 (M/s TA Instruments, MI, DE, USA). A sample of 1–2 mg was crimped in the aluminum pans and heated from 25°C to 350°C at a scanning rate of 10°C per minute under an air flow of 100 ml/min. Empty aluminum pans were used as the reference. The thermograms were recorded using Platinum™ software [36].

### FTIR spectroscopy

An FTIR spectrum of the pure Mgf was obtained using an FTIR-8300 spectrophotometer (Shimadzu, Japan). A total of 2% (w/w) of sample, with respect to the KBr disk, was mixed by trituration to obtain the fine powder and subsequently compressed to form a disk using a hydraulic press at 10,000 psi for 30 s. Each KBr disk was scanned at 4 mm/s at a resolution of 2 cm over a wave number region of 4000–400 cm^-1^ using IR solution software (version 1.10) [35].

### Scanning electron microscopy

The scanning electron microscopy (SEM) studies were performed to visualize the surface morphology of Mgf. Samples were mounted on aluminum stubs using double-sided adhesive tape and sputter coated with a thin layer of gold at 10 Torr vacuum before examination (JEM-2100F, M/s Jeol, Tokyo, Japan). Mgf was coated with platinum (1:1) in a sputter coater and their surface morphology was viewed and photographed [36].

## Results & discussions

### Preparation of calibration curve

Linearity of the developed method was determined by analyzing serial dilutions of Mgf between a concentration range of 200 and 1000 ng/band, respectively, and plotting the peak height versus concentration to obtain a linear correlation plot at 262 nm with R_f_ = 0.68 ± 0.02. The linearity of the equation was observed to be Y = 7.686× with the coefficient of correlation (R) as 0.997 (p < 0.001) [10]. The system suitability parameters were determined to validate the robustness of the method and were found to be within permissible limits as per the International Conference on Harmonisation Q2 guidance (R1) [37] (data have been shown in [24]).

### Evaluation of physicochemical properties

The experimental results have been compiled in Table 2 in order to compare the predicted and observed values, and to find any statistical difference between them. The results clearly depict that Mgf exhibited poor solubility in water and fasted-state simulated gastric fluid, while it showed relatively moderate solubility in fasted-state simulated intestinal fluid and appreciable solubility in fed-state simulated intestinal fluid. The increased solubility in gastrointestinal and intestinal milieu can primarily be attributed to the presence of surfactants [38].

The values of two other important physicochemical lipophilicity descriptors, *viz.*, log P and log D, were also reported to be quite low, accounting for poor permeability. The results are in close consonance with the low values of effective permeability observed during single pass intestinal perfusion studies [11].

On the basis of the low values of solubility in various aqueous media and values of lipophilicity observed for Mgf, it could be safely regarded as a candidate belonging to biopharmaceutical classification system (BCS) class IV, a fact aptly supported by some of the previous scientific reports too [43,44].

Statistical significance of difference was discerned among predicted, experimental and reported values for various parameters using Student's independent *t*-test. High level of statistically significant difference among aqueous solubility values could plausibly be due to variation in the experimental conditions such as pH of water, temperature and technique employed for analysis, and varying botanical sources of Mgf used by other scientists [45–47]. Very significant difference between the permeability parameters, however, can be attributed on the basis of the values predicted using software *vis-à-vis* those observed experimentally in rats.

### P-gp efflux assay

Overexpression of P-gp in Caco-2 cells is well documented in the literature [48]. P-gp confers resistance by preventing enough accumulation of drugs within the cell, thereby preventing their cytotoxic or apoptotic effects [49]. This is achieved by its ability to mediate ATP-dependent drug translocation across the plasma membrane against considerable concentration gradient. The P-gp efflux assay revealed that no transport of dyes was observed at 37°C as the MDR1 and BCRP transporters were active and effluxed the dyes through pumps, resulting thereby in less than 20% fluorescence intensity (Figure 2). On the other hand, incubation of dyes with vinblastine showed that at 37°C, the latter may block both the MRP1 and BCRP transporters, eventually leading to higher fluorescence intensity (more than 80%), as no efflux was encountered [33]. On the contrary, Mgf loaded with Rh-123 and DiOC_2_ showed less than 20% fluorescence. Being a P-gp substrate, this can be ascribed to easy efflux of Mgf and dyes by P-gp pumps, resulting eventually in drastic reduction of fluorescence intensity inside the Caco-2 cells [16].

### Spectroscopic, thermoanalytical & microscopic characterization

In order to standardize and characterize Mgf for its physical state and surface morphology, the P-XRD, DSC, FTIR and SEM studies were performed. The P-XRD spectrum of Mgf showed sharp peaks indicating its crystalline nature (Figure 3A). The DSC thermogram of Mgf indicated a sharp endothermic peak at 277.08°C, ratifying its crystalline nature (Figure 3B). The FTIR studies of Mgf revealed functional groups at peaks at 3399 cm^−1^ (O–H_str_), 2932.8 cm^−1^ (aliphatic C–H_str_), 1636.75 cm^−1^ (C=O_str_), 1497.10 cm^−1^ (CH–CH_str_) and 1253.00 cm^−1^ (C–O_str_) cm^−1^. Particularly, the characteristic peak at 1023.22 cm^−1^ indicated the presence of C–C stretching in the Mgf structure (Figure 3C). During SEM studies too, Mgf showed longitudinal column shape crystals, as depicted in Figure 3D.

## Conclusion

Physicochemical characterization of a bioactive is an essential prerequisite in order to formulate systems for its effective delivery. As the existing literature on the physicochemical properties of Mgf is quite limited, endeavors were made to determine and report the physicochemical properties. The current research article enlightens the vital physicochemical properties of this bioactive, thus furnishing scope for developing and designing newer drug delivery strategies offering its maximal bioactivity. The values reported in the work could further facilitate scientists to develop newer delivery systems for this promising BCS class IV bioactive, demonstrating diverse medicinal activities and therapeutic promise. In conclusion, it is intended that this manuscript be a steering guide for the drug industry and research groups to explore this potential bioactive.

## Future perspective

These reported data on physicochemical aspects of Mgf are likely to pave the way for effective development of potential nanostructured systems for investigating the mechanism(s) of drug interactions with biological macromolecules. Nevertheless, a lot of information still needs to be explored, especially about its biopharmaceutical and pharmacokinetic aspects.

**Table 1.** **Results of *in silico* simulations on mangiferin.**

**Property/parameter**	**Value**
Structure	
IUPAC name	(1S)-1,5-Anhydro-1-(1,3,6,7-tetrahydroxy-9-oxo-9H-xanthen-2-yl)-D-glucitol
Molecular formula	C_19_H_18_O_11_
Molecular weight	422.35
Solubility in water	1.44 mg/ml
Solubility fasted-state simulated gastric fluid	0.133 mg/ml
Solubility fasted-state simulated intestinal fluid	1.51 mg/ml
Solubility fed-state simulated Intestinal fluid	4.67 mg/ml
Melting point	274°C
log P	-0.59
log D (pH 7.4)	-0.80
P_eff_	0.27 × 10^-4^ cm/s
P-gp substrate	Yes

Predicted values are obtained from GastroPlus^TM^ software.

log P: partition coefficient; log D: distribution coefficient; P_eff_: effective permeability.

**Table 2.** **Observed physicochemical properties of mangiferin.**

**Parameter**	**Predicted values from GastroPlus^®^**	**Experimental value**	**p-value**	**Reported values from literature**	**Ref.**
Aqueous solubility	1.44 mg/ml	0.38 mg/ml at 25°C	< 0.001^†^	0.25 mg/ml at 45°C	[39]
Fasted-state simulated gastric fluid	0.133 mg/ml	0.140 mg/ml at 25°C	> 0.05 (NS)	Not reported in literature	–
Fasted-state simulated intestinal fluid	1.51 mg/ml	1.48 mg/ml at 25°C	> 0.05 (NS)	Not reported in literature	–
Fed-state simulated intestinal fluid	4.67 mg/ml	3.98 mg/ml at 25°C	< 0.001^†^	Not reported in literature	–
Melting point	–	274°C	–	270°C	[40]
log P	-0.59	-0.65	> 0.05 (NS)	-0.09 to -0.36	[41]
log D	-0.80	-0.79	> 0.05 (NS)	Not reported in literature	–
P_eff_	0.27 × 10^-4^ cm/s	0.16 × 10^-4^ cm/s	< 0.01^‡^	Not reported in literature	–
P-gp substrate	Yes	Yes	–	Yes	[14,42]

Observed values are mean of three values.

^†^Appreciable significant difference.

^‡^Significant difference.

log P: partition coefficient; log D: distribution coefficient; NS: Not significant; P_eff_: Effective permeability.

**Executive summary**

The current research was undertaken to reveal the physicochemical properties of mangiferin (Mgf) through GastroPlus™ software.
Mgf encounters numerous challenges when administered orally, principally due to its poor solubility, low bioavailability, high hepatic first-pass metabolism and high P-gp efflux. Looking into these challenges, the need of the hour is to incorporate Mgf into a delivery system that has distinct potential to improve its bioavailability and, eventually, increased drug levels into systemic circulation.
The present research work endeavours to investigate various aspects of Mgf such as its equilibrium solubility in water, simulated gastric fluid and simulated intestinal fluid, melting point, lipophilicity as logarithm of octanol–water partition coefficient and tendency to act as a P-gp substrate, estimated through the ‘*in silico*’ approach employing GastroPlus^®^ software. Mgf has also been characterized through transmission electron microscopy, differential scanning calorimetry, Fourier transform infrared and powder x-ray diffraction studies.
Poor solubility was observed in water whereas increased solubility in gastrointestinal and intestinal milieu can primarily be attributed to the presence of surfactants. log P and log D were also reported to be quite low, accounting for poor permeability and ratifying that Mgf is a BCS class IV molecule.
P-gp efflux assay revealed in to be a potent P-gp substrate.
These physicochemical values reported for Mgf in the work would further facilitate the scientists to develop newer delivery systems for this BCS class IV molecule, demonstrating diverse medicinal activities.
Based on the provided *in silico* and observed data, a better preview of the pharmacokinetic parameters of Mgf can be explored, in clinical trials.
